# Western Iowa Veterinary Medical Association

**Published:** 1891-12

**Authors:** G. A. Johnson

**Affiliations:** Secretary


*Western Iowa Veterinary Medical Association.*—The third meeting of the Western Iowa Veterinary Medical Association was held October 21st, 1891. The meeting was called to order by President Johnston. Present: S. H. Johnston, President, J. I. Gibson, Vice-President, G. A. Johnson, Secretary-Treasurer, and Prof. W. B. Niles, of the Veterinary Department of the Iowa Agricultural College.

Minutes of previous meeting read and approved. Letters were read from J. J. Miller, V. S., Sioux City, S. Stewart, D. V. M., Council Bluffs, and J. M. Smith, V. S., Cherokee. The President reported that as a committee to procure a list of service fees, of the Ontario Veterinary College, and I. S. M. D. A. he had secured a list from the college but not the other.

Under unfinished business, two articles, one on ethics, and one regulating changes of the by-laws were added and the by laws thus completed were adopted. Under new business, the subject of legislation was discussed and it was the unanimous opinion that it would be impossible to get an iron clad bill through the coming legislature, and that any law that recognizes quacks, who have just been practicing five or ten years is a detriment, and an insult to the profession. But all thought that a law could be framed and passed, that puts no restrictions on quacks practicing, but forbids them the use of the title Veterinarians or analogous titles. If such can be had, it will enlighten the public, so that they will know what kind of a practitioner they are employing. A list of service fees were drafted to be presented to the I. V. M. A. at the meeting to be held at Des Moines, November 12 and 13, 1891.

The Secretary was elected a delegate to the I. S. V. M. A. The application of J. J. Miller. V. S., of Sioux City, was presented and he was elected a member.

The President then called on G. A. Johnson who presented a paper on "The Preparation of Animal Sutures and Pilocarpine as a Purgative for the Horse."

During the discussion that followed Prof. Niles advanced the idea that eserine in large doses caused the discharge of watery fæces.

Prof. Niles then reported a case, from the college hospital record, of pelvic abscess situated between the rectum and the vagina, which was opened per vagina, and readily healed under antiseptic treatment.

The association then adjourned to meet again in January, 1892.

G. A. Johnson, *Secretary.*

